# Proteomics of autism and Alzheimer’s mouse models reveal common alterations in mTOR signaling pathway

**DOI:** 10.1038/s41398-021-01578-2

**Published:** 2021-09-17

**Authors:** Shira Mencer, Maryam Kartawy, Felix Lendenfeld, Huda Soluh, Manish Kumar Tripathi, Igor Khaliulin, Haitham Amal

**Affiliations:** Institute for Drug Research, School of Pharmacy, Faculty of Medicine, The Hebrew University of Jerusalem, Jerusalem, Israel

**Keywords:** Molecular neuroscience, Diagnostic markers

## Abstract

Autism spectrum disorder (ASD) and Alzheimer’s disease (AD) are two different neurological disorders that share common clinical features, such as language impairment, executive functions, and motor problems. A genetic convergence has been proposed as well. However, the molecular mechanisms of these pathologies are still not well understood. Protein S-nitrosylation (SNO), the nitric oxide (NO)-mediated posttranslational modification, targets key proteins implicated in synaptic and neuronal functions. Previously, we have shown that NO and SNO are involved in the InsG3680(+/+) ASD and P301S AD mouse models. Here, we performed large-scale computational biology analysis of the SNO-proteome followed by biochemical validation to decipher the shared mechanisms between the pathologies. This analysis pointed to the mammalian target of rapamycin complex 1 (mTORC1) signaling pathway as one of the shared molecular mechanisms. Activation of mTOR in the cortex of both mouse models was confirmed by western blots that showed increased phosphorylation of RPS6, a major substrate of mTORC1. Other molecular alterations affected by SNO and shared between the two mouse models, such as synaptic-associated processes, PKA signaling, and cytoskeleton-related processes were also detected. This is the first study to decipher the SNO-related shared mechanisms between *SHANK3* and *MAPT* mutations. Understanding the involvement of SNO in neurological disorders and its intersection between ASD and AD might help developing an effective novel therapy for both neuropathologies.

## Introduction

Autism spectrum disorder (ASD) is a neurodevelopmental disorder associated with impaired social skills, restricted/repetitive behaviors, and communication deficits [1]. Patients may display co-occurring symptoms like seizures, anxiety, and attention deficits hyperactivity disorder [2]. ASD is prevalent in ~1.5% of the population [3] and dramatic growth in the prevalence rates of this disorder has been reported over the last few decades [4]. The increase in the number of people with ASD has led to a very significant negative impact on the economy. In the US alone, the economic burden of this disorder was estimated at $268 billion in 2015 [5]. Existing treatments offer little benefit for the core symptoms, making the search for novel treatment options necessary [6].

Alzheimer’s disease (AD) is a neurodegenerative disease that comprises up to 80% of all dementias and affects 10% of the population aged 65 and older. Characteristic symptoms that severely impair the ability to perform everyday activities at the late stage of the disease include the progressive loss of memory, decline of cognitive skills, and deterioration of speech [7]. Despite global efforts to identify treatments against this pathology, only the treatments able to alleviate some symptoms of AD are currently available. Recognizing the need for additional therapeutic strategies against AD, the World Health Organization has made this disease a public health priority [8].

The two hallmark features defining AD are neurofibrillary tangles representing aggregates of twisted strands of hyperphosphorylated tau protein, and amyloid plaques mainly made up of accumulations of amyloid-β (Aβ) peptides [7]. Proteolytic cleavage of the mature Aβ protein precursor (APP) by β- and γ-secretase via the amyloidogenic pathway generates neurotoxic Aβ peptides consisting of 40 and 42 amino acids, which aggregate to make up the main components of neurotic plaques resulting in brain atrophy in individuals with AD [9]. Alternative, non-amyloidogenic processing of APP by α- and γ-secretase, produce the secreted α-form of APP (sAPPα), which is generally recognized as neurotrophic and neuroprotective [10]. These properties of sAPPα have made it a target for the treatment of neurodegenerative diseases such as AD [11, 12]. Interestingly, in addition to age-related neurodegenerative diseases, tau and the amyloid-β protein precursor (APP) have also been shown to play a role in neurodevelopmental disorders such as autism [6, 13–15]. Thus, it has been found that the level of sAPPα is elevated in severe (but not mild or moderate) autism [13–15]. It is suggested that promotion of the neurotrophic non-amyloidogenic pathway resulting in sAPPα accumulation in autistic patients leads to early megalencephaly, causing interneuronal misconnections potentially underlying a number of autism-related symptoms [16].

Hyperphosphorylation of tau that presumably causes neurodegeneration in AD patients [17] may also play a role in ASD pathogenesis. Thus, Tai et al. have shown in two distinct mouse models of ASD that a 50% tau reduction is sufficient to prevent or significantly diminish autism-like behaviors, megalencephaly, and epilepsy co-occurring in ASD subjects [6]. The study also found that a reduction of tau increased the activity of PTEN, a suppressor of the PI3K/Akt/the mammalian target of rapamycin (mTOR) signaling pathway. The PI3K/Akt/mTOR pathway is commonly overactivated in ASD [18–20], and the authors postulated that various symptoms of ASD caused by aberrant activation of this signaling pathway could be counteracted by a reduction in tau levels [6].

Another link between AD and ASD has been established by Gozes and colleagues [21–23]. Activity-dependent neuroprotective protein (ADNP) regulates more than 400 genes during neurodevelopment and is essential for brain formation and neurogenesis [23]. De novo mutations of ADNP resulted in developmental delays and intellectual disabilities, including motor and speech dysfunctions [24, 25], and are estimated to occur in at least 0.17% of all ASD cases, making it one of the most common genes implicated in autism [22, 23]. Meanwhile, ADNP deficiency has been reported to promote tauopathy in AD patients [23, 26, 27]. Furthermore, a number of ADNP variants have been discovered in the brains of postmortem AD patients, leading to the hypothesis that ADNP mutations also affect the aging brain and neurodegeneration when mutated in a mosaic fashion [21].

Increasing attention is being drawn to the role of S-nitrosylation (SNO) in neuropathology. Protein SNO is a posttranslational modification by which nitric oxide (NO) is covalently attached to a cysteine thiol of a protein to form an S-nitrosothiol leading to major implications for the functions and downstream signal transduction of this protein [28]. Recently, we have mapped the SNO-proteome in two different mouse models, an ASD model based on InsG3680(+/+) mutation of the *Shank3* gene [29] and an AD model based on P301S mutation causing increased tau phosphorylation [30]. The P301S mutation in the human tau protein encoded by the *MAPT* gene is linked to frontotemporal dementia (https://www.cell.com/fulltext/S0896-6273(07)00030-X). Our experiments on the *Shank3* model of ASD indicated that the InsG3680(+/+) mutation alters SNO-proteome, resulting in an enrichment of certain pathways and processes linked to ASD pathology [29]. Meanwhile, our experiments on the P301S AD mouse model have also revealed major changes in the SNO-proteome in the cortex suggesting that the SNO of proteins contributes to tau pathology through regulation of calcium and non-canonical Wnt signaling [30]. We suggest that ASD and AD pathogenesis may involve common SNO-related mechanisms.

**Fig. 1:**
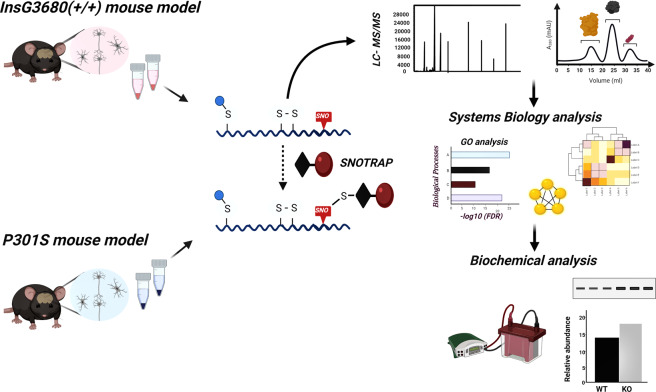
Schematic workflow of the study. Schematic workflow of the SNOTRAP-based MS analysis of the ASD and AD cortex samples followed by large-scale systems analysis and biochemical validation.

In this study, we conduct a comparative analysis of the two models (the *Shank3* model of ASD [31] and the P301S model of AD https://www.sciencedirect.com/science/article/pii/S089662730700030X) to investigate the shared biological processes (BP) and pathways that might be affected by aberrant SNO signaling in neurodevelopmental and neurodegenerative disorders. For this purpose, we used SNOTRAP-based mass spectrometry (MS) technology [29] followed by systems biology analysis combined with bioinformatics. The key shared proteins were validated biochemically, see schematics workflow of this study in Fig. 1.

## Materials and methods

### Materials, reagents, and animals

Vivapsin 10 kDa molecular weight cut-off (MWCO) filters were procured from Sartorius AG (Germany). For MS, acetonitrile (ACN) and distilled water were purchased from Sigma-Aldrich (St. Louis, MO). HPLC grade solvents were used for high-performance liquid chromatography (HPLC) and liquid chromatography-MS (LC-MS). Biotin-PEG3-propionic acid was derived from Chem Pep Inc. (Florida, USA). Sequencing-grade modified trypsin was provided by Promega (Wisconsin, USA). SNOTRAP-biotin synthesis and nuclear magnetic resonance analysis were performed as described previously [32].

All methods were carried out in accordance with the Hebrew University guidelines and regulations. Animal data previously generated were taken from the Pride Software mentioned below. All animal experiments were conducted in accordance with the Institutional Animal Care and Use Committee and the Association for Assessment and Accreditation of Laboratory Animal Care International. Mice were purchased from the Jackson laboratory. The juvenile male InsG3680(+/+) mice harbor the ASD patient-linked single guanine nucleotide (G) insertion at cDNA position 3680, which leads to a frameshift and downstream premature stop codon. The juvenile male tau P301S mice harbors the T34 isoform of microtubule-associated protein tau with one N-terminal insert and four microtubule-binding repeats (1N4R) encoding the human P301S mutation.

### Brain tissue sample preparation for MS

All samples were prepared in the dark at room temperature. Cortex tissues were isolated from juvenile (6–8 weeks) ASD, AD, and WT mice following decapitation during the daytime as described previously [33]. The brain samples were immediately transferred into liquid nitrogen and stored at −80 °C for further analysis. Per each of the biological replicate, three cortex tissue samples from three mice were pooled. Two biological replicates each containing three technical replicates were run. Further, tissues were homogenized on ice in freshly prepared lysis buffer containing: 250 mM HEPES-NaOH, 0.1 mM neocuproine, 1 mM EDTA, 1% NP-40, 20 mM iodoacetamide (IAM), 1% protease inhibitors cocktail, pH 7.7. The homogenates were centrifuged (12,000–13,000*g* for 10 min at 4 °C), the supernatant was collected, and protein concentration was estimated by Bradford assay (Bio-Rad, California USA, Cat. No. 500-0006). Next, in the presence of 2.5% SDS, samples were alkylated with 30 mM IAM in the dark at 37 °C. After alkylation, samples were washed twice with three times volume of 8 M urea (in 50 mM HEPES, pH 7.7) and once with 50 mM HEPES (pH 7.7) by centrifugation at 5000*g* for 30 min at 4 °C with 10K MWCO spin filters pre-rinsed once with water (Sartorius AG, Germany, Cat. No. VS15T01). After the centrifugation, SNOTRAP-labeling stock solutions (in 50% ACN) were added to all samples to reach a final concentration of 1.25 mM. This was performed with the purpose of converting SNO to stable disulfide-iminophosphorane. Further, at 25 °C, all samples were incubated for 1.5 h in SNOTRAP solution. Succeeding the SNOTRAP labeling, the reagents were removed by three consecutive washing with 50 mM HEPES (pH 7.7) buffer with 10 K filters. Post ultrafiltration, each sample was incubated with 200 μl pre-rinsed Streptavidin agarose beads (Pierce, Cat. No. 20349) for 1 h at room temperature with gentle shaking. The beads were washed with washing buffer (50 mM HEPES, 150 mM NaCl, 0.1% SDS, pH 7.7) three times and then with another washing buffer (50 mM HEPES, pH 7.7) three times. Following washing, proteins were eluted with the buffer containing: 10 mM TCEP in 50 mM HEPES, pH 7.7, and then alkylated with 10 mM IAM. Protein samples were then trypsinized (Promega, Wisconsin, USA, Cat. No. V5111) at 37 °C for 4 h and then desalted with C18 StageTips as described previously [34].

### Analysis flowchart of MS

The digested peptides were analyzed using the 6550 Nano-HPLC-Chip/MS system of Agilent coupled with a micro-autosampler, pumps of a capillary and nanoflow, the Chip-Cube connected to the LC modules, and the MS instrument. H_2_O with 0.1% formic acid (FA) was used as a mobile phase A and ACN with 0.1% FA was used as a mobile phase B. Polaris-HR-Chip-3C18 HPLC-Chip (Agilent Technologies, Cat. No. G4240-62030) separated the peptides. It consisted of a 360 nl enrichment column, a 75 μm × 150 mm analytical column, and a 3 μm stationary phase. The peptides were loaded into the enrichment column. The gradient was set for 55 min, starting from 3% B at 300 nl/min, increased to 30% B, and kept from the 2nd to 35th min, then increased to 60% B at the 40th min, to 90% B at the 45th min and then kept stable for 5 min followed by a 5 min after-run at 3% B. We acquired the positive-ion MS spectra using 1700 Da extended dynamic range mode: electrospray ionization ESI capillary voltage was set on 1960 V; fragmentor on 360 V; Octopole RF peak on 750 V; drying gas on 13 l/min; drying temperature on 225 °C. The data were acquired at the rate of 6 MS spectra/sec and 3 MS/MS spectra/s in the range of *m*/*z* 300 to 1700 for MS and 50 to 1700 for MS/MS. The max number of precursors per cycle was set at 20, setting the threshold at 5000 ions in a precursor abundance-based scan speed in peptide isotope model with plus 2, plus 3, and above charge-state preference, and with active exclusion after one spectrum and released after 0.15 min. The fragmentation energy was set at a slope of 3.1 V/100 Da, including a 1.0 offset for doubly charged precursors, 3.6 V/100 Da with a −4.8 offset for triply and also multiply charged precursors. We used Agilent MassHunter Workstation software for the data acquisition. The mass accuracy was preserved using ion *m*/*z* 1221.9906 as an internal reference.

### Processing of the MS data

For peak list generation, database searching, and false discovery rate (FDR) estimation, Agilent Spectrum Mill MS proteomics Workbench B.05 software was used. The following parameters for data extractions were conducted: precursor MH+ 300–8000 Da, scan time range from 0 to 200 min, a sequence tag length of >1, default for precursor charge, true for find 12C precursor, merge scans with the same precursor at ±30 s and ±0.05*m*/*z*, and a MS noise threshold of 100 counts. MS/MS spectra were searched against the mouse SwissProt protein database with ±20 ppm precursor ion tolerance and ±50 ppm fragment ion tolerance. Different modifications of methionine oxidation, deamidation of asparagine, and a fixed modification of cysteine carbamidomethylation were included. The generated FDR was set at 1.2% for both peptide and protein identification. The MS proteomics data used in this study, which we generated previously, were taken from ProteomeXchange Consortium database (http://proteomecentral.proteomexchange.org) via the PRIDE partner repository with the dataset identifier <PXD006907> for ASD data and <PXD010106> for AD data.

### Statistics and systems biology analysis

For the systems biology analysis of the BP and pathways analysis, we uploaded the lists of all SNO proteins into MetaCore from Clarivate Analytics (MetaCore V6.34 build 69200 software). The Benjamini–Hochberg correction [35] was used to calculate the *P* value and generate FDR. Terms with FDR values below 0.05 were accepted. The search tool for the interacting proteins (STRING, version 10.0) was used to analyze the protein–protein interaction of SNO proteins (http://string-db.org) [36]. Strong reliability interactions (score > 0.7) from the neighborhood, gene fusion, co-occurrence, co-expression, experiments, databases, and text mining lists were used. Cytoscape V3.3.0 software was used for visualization of the protein–protein interaction. MetaCore from Clarivate Analytics (MetaCore V6.34 build 69200 software) was used for the network generation after submitting the lists of SNO proteins. For this purpose, we also used Benjamini–Hochberg correction to calculate the *P* value and generate FDR. The processes/terms with the FDR values below 0.05 were included. GraphPad PRISM 8 software was used to generate the schematic figure and heat map.

### Western blot

#### Protein extraction and estimation

The cortex tissue was homogenized and sonicated in RIPA buffer (Sigma-Aldrich, USA, Cat. No. R0278) containing protease and phosphatase inhibitors cocktail, centrifuged at 4 °C, and the supernatant was collected. Protein content was measured in the supernatant using the BCA (Sigma-Aldrich, USA, Cat. No. B9643) method.

#### WB analysis

The protein content in the samples was estimated and then subjected to polyacrylamide gel electrophoresis (Bio-Rad #1610185), followed by wet transfer onto a polyvinylidene fluoride (PVDF) membrane (Bio-Rad #1620177). Non-specific-binding sites were blocked by 5% BSA in tris-buffered saline (135 mM NaCl, 2.5 mM KCl, 50 mM Tris, and 0.1% Tween 20, pH 7.4) for 2 h at room temperature (RT). PVDF membrane with the transferred proteins were incubated with the primary antibody of anti-p-RPS6 (dilution 1:1000; Cell Signaling Technology, #4858), anti-RPS6 (dilution 1:1000; Cell Signaling Technology #2317), anti-beta-actin (dilution 1:1000; Cell Signaling Technology #3700) overnight at 4 °C on a shaker. After the incubation with the primary antibodies, the membrane was washed with TBST buffer and incubated with anti-mouse/rabbit horseradish peroxidase-conjugated secondary antibody for 1 h at RT. Specific binding of the protein of interest was detected using ECL substrate (Bio-Rad #1705062). The bands were visualized using a Bio-Rad Chemidoc imaging system.

## Results

### BP analysis of the SNO-proteome in ASD and AD mouse models

**Fig. 2:**
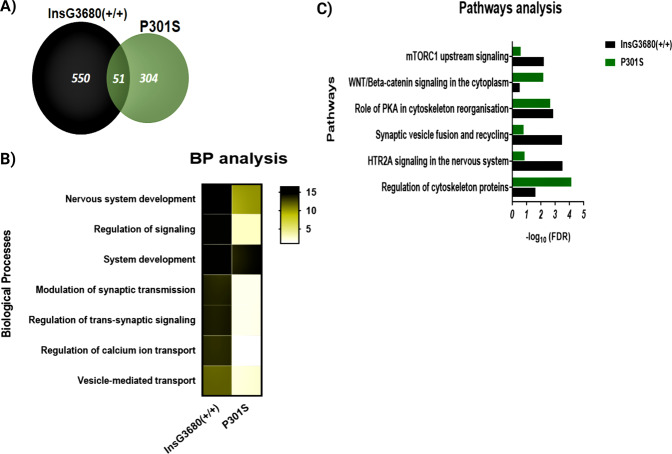
Systems biology analysis of the SNO proteins of the ASD and AD cortex samples. **A** Venn diagram of the SNO proteins. **B** Heat map representing the BP analysis conducted on the SNO proteins exclusive to ASD and AD models. *The scale represents the −log10 of the corrected false discovery rate (FDR). **C** Pathways analysis of the SNO proteins exclusive to ASD and AD models. *Bars represents the −log10 of the Benjamini corrected false discovery rate (FDR).

Proteomic analysis using the SNOTRAP-based MS tool revealed 550 proteins that are exclusive to ASD mouse model, 304 proteins exclusive to AD, and 51 proteins shared between the two groups. See the Venn diagram in Fig. 2A. Table 1 shows the list of SNO proteins shared between the two models. The detailed lists of the SNO proteins in both groups are presented in Supplementary Table 1.

**Table 1:** The shared SNO-proteins between ASD and AD models.

Accession ID	Protein’s name
P06837	Neuromodulin
P34884	Macrophage migration inhibitory factor
Q7SIG6	Arf-GAP with SH3 domain, ANK repeat and PH domain-containing protein 2
Q6R5N8	Toll-like receptor 13
Q6PIX9	Uncharacterized protein C17orf80 homolog
A2AF47	Dedicator of cytokinesis protein 11
Q8BLQ9	Cell adhesion molecule 2
Q6ZQA0	Neurobeachin-like protein 2
Q8BXQ2	GPI transamidase component PIG-T
Q5QNQ9	Collagen alpha-1 (XXVII) chain
Q3UR32	P2X purinoceptor 3
Q9JJ26	Pyrin
Q6XQH0	Galactose-3-*O*-sulfotransferase 2
Q6RHR9	Membrane-associated guanylate kinase, WW and PDZ domain-containing protein 1
P41234	ATP-binding cassette sub-family A member 2
Q8CFA1	Interleukin-1 receptor-associated kinase-like 2
Q8C9B9	Death-inducer obliterator 1
A2AAE1	Uncharacterized protein KIAA1109
Q8BMS1	Trifunctional enzyme subunit alpha, mitochondrial
Q9Z329	Inositol 1,4,5-trisphosphate receptor type 2
Q8BI84	Melanoma inhibitory activity protein 3
Q9D5V5	Cullin-5
Q6PFX7	Neuronal tyrosine-phosphorylated phosphoinositide-3-kinase adapter 1
Q8BUV3	Gephyrin
E9Q557	Desmoplakin
Q02257	Junction plakoglobin
Q9CQ48	NudC domain-containing protein 2
Q9WUS4	Gap junction alpha-10 protein
Q9D4K4	FANCD2 opposite strand protein
O35166	Golgi SNAP receptor complex member 2
O55047	Serine/threonine-protein kinase tousled-like 2
Q7TT50	Serine/threonine-protein kinase MRCK beta
Q80X90	Filamin-B
A2AU72	Armadillo repeat-containing protein 3
Q06890	Clusterin
O88455	7-Dehydrocholesterol reductase
Q587J6	LINE-1 type transposase domain-containing protein 1
Q3V1U8	ELMO domain-containing protein 1
Q6PDI5	Proteasome-associated protein ECM29 homolog
Q06335	Amyloid-like protein 2
Q8BGQ7	Alanine–tRNA ligase, cytoplasmic
Q8C779	Uncharacterized protein CXorf57 homolog
Q9QZE7	Translin-associated protein X
Q9QZQ1	Afadin
Q91Y44	Bromodomain testis-specific protein
Q8BUH8	Sentrin-specific protease 7
Q3UQ44	Ras GTPase-activating-like protein IQGAP2
Q8CHY6	Transcriptional repressor p66 alpha
Q6PGA0	REST corepressor 3
Q00558	Factor VIII intron 22 protein
Q91ZU6	Dystonin

The BP analysis was performed to identify the biological mechanisms that are modulated by SNO in both models. This analysis demonstrated significant enrichment of synaptic and neuronal processes that appeared to be common to both *Shank3* and P301S models. Thus, modulation of chemical synaptic transmission (false discovery rate (FDR) = 1.10E−14 in ASD, FDR = 1.13E−02 in AD), regulation of trans-synaptic signaling (FDR 1.20E−14 in ASD, FDR = 1.16E−02 in AD), nervous system development (FDR = 3.21E−17 in ASD, FDR = 4.86E−11 in AD), and others have found to be enriched (Fig. 2B).

### Pathways analysis of the SNO-proteome in ASD and AD mouse models

Pathways analysis of the SNO proteins that are exclusive to ASD and exclusive to AD revealed common enrichment of pathways affected in both models including WNT/beta-catenin signaling in the cytoplasm (FDR = 6.72E-03), HTR2A signaling in the nervous system (FDR = 3.00E-04), the role of PKA in cytoskeleton organization (FDR = 1.37E-03), and others (Fig. 2C).

Importantly, our analysis revealed the involvement of the SNO proteins in the mammalian target of rapamycin complex 1 (mTORC1) upstream signaling pathway (FDR = 5.98E-03). A subset of proteins that are associated with both ASD and AD showed to be involved in the mTOR signaling pathway. These include RAC1, WNT11, Frizzled10 in AD and TSC2, P38 MAPK, AGTR1, PDGF receptor, mLST8, and insulin receptor in ASD mice (Fig. 2C). Supplementary Table 2 summarizes the systems biology analysis of both models.

### Interactome and clustering analysis of the SNO proteins

**Fig. 3:**
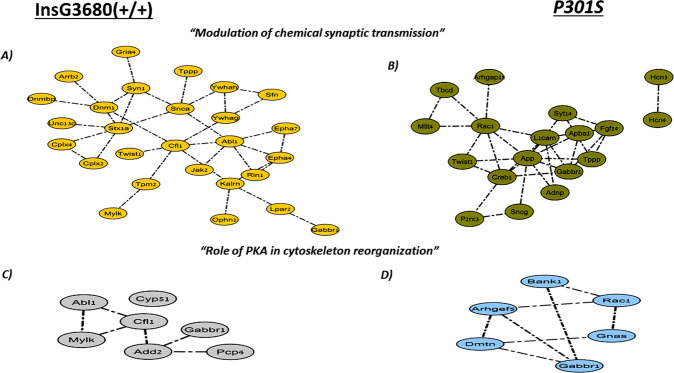
Clustering analysis of the SNOed proteins in ASD and AD mouse models. “Modulation of chemical synaptic transmission” cluster was enriched in **A** ASD and **B** AD, and the “role of PKA in cytoskeleton reorganization” cluster was enriched in **C** ASD and **D** AD.

The proteins were classified into clusters based on their enriched biological processes and pathways. Different subsets of SNO proteins exclusive to ASD and AD formed distinct clusters belonging to the same biological processes and pathways that are suggested to contribute to the pathogenesis of ASD and AD. The yellow-ASD and green-AD nodes in Fig. 3 correspond to the SNO proteins that are involved in “Modulation of chemical synaptic transmission”, including SYN1, STX1A, JAK2, and others in ASD (Fig. 3A) and TBCD, RAC1, P2RX3, SYT14, and others in AD (Fig. 3B). Furthermore, the involvement of PKA in cytoskeleton reorganization was found to be common to both models as well. The gray-ASD nodes included CYP51, CFI1, ADD2, and others (Fig. 3C) and the blue-AD nodes included BANK1, GNAS, GABBR1, and others (Fig. 3D).

### Biochemical analysis of mTORC1 pathway

**Fig. 4:**
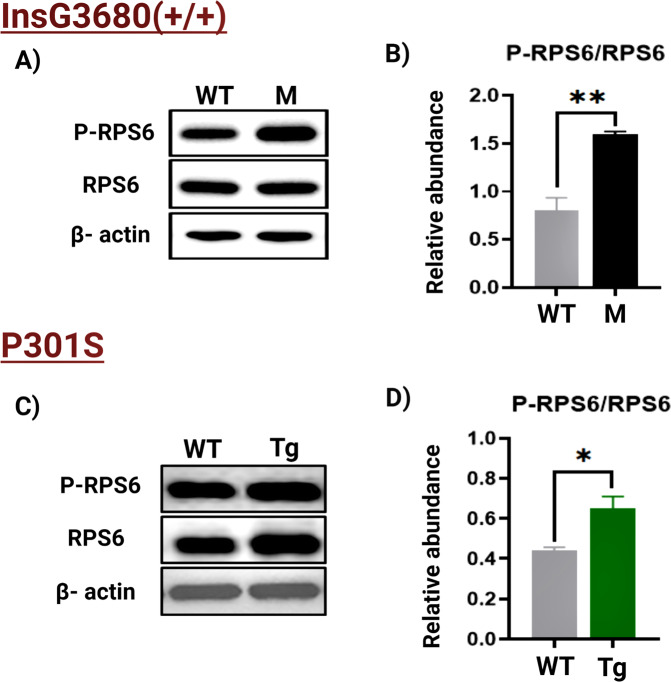
WB analysis. **A** Representative WB of RPS6 and P-RPS6 prepared from the cortex tissues from the WT mice (*n* = 5) and ASD mouse model (abbreviated with M; *n* = 5). **B** The relative average WB intensity of P-RPS6, showing a significant increase in the phosphorylation levels of the RPS6 in mutant mice compared to the WT. The data are normalized to RPS6 and beta-actin presented as mean ± SEM. A two-tailed *t*-test was conducted. **P < 0.01. **C** Representative WB of RPS6 and P-RPS6 prepared from the cortex tissues from the WT mice (*n* = 5) and AD mouse model (abbreviated with Tg; *n* = 5). **D** The relative average WB intensity of P-RPS6, showing a significant increase in the phosphorylation levels of the RPS6 in Tg mice compared to their WT littermates. The data are normalized to RPS6 and beta-actin presented as mean ± SD. A two-tailed *t*-test was conducted. *P < 0.05.

**Fig. 5:**
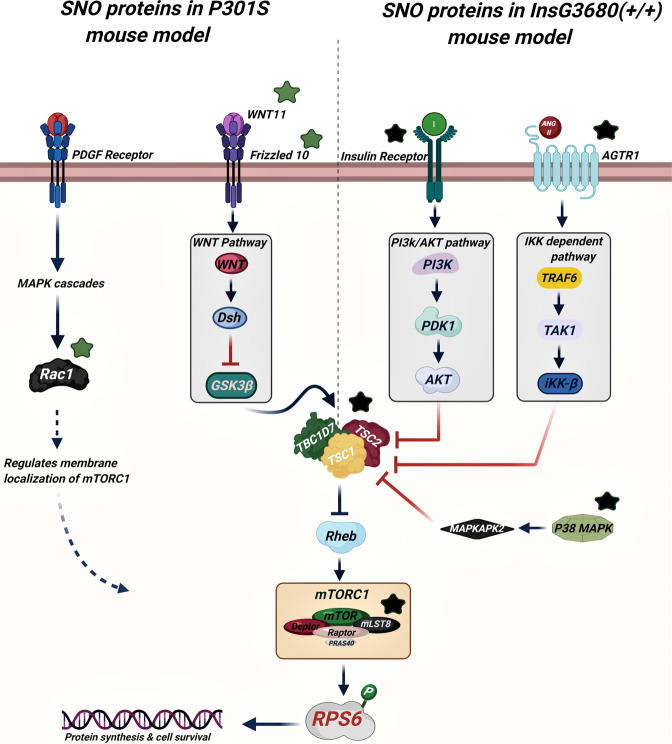
The suggested scheme of the SNO-dependent mTOR activation in the ASD and AD mouse models. Green stars are SNO proteins in AD*.* Black stars are SNO proteins in ASD.

To validate our bioinformatics analysis and test whether the mTORC1 signaling pathway is altered in both models, we quantified the phosphorylation levels of a major substrate of the mTORC1, ribosomal protein S6 (RPS6). In ASD, WB analysis showed significantly elevated levels of the phospho-RPS6 (P-RPS6) in the mutant ASD mice compared to their wild-type (WT) counterparts (Fig. 4A, B). A significant increase of P-RPS6 was also observed in the mutant AD mice compared to the WT (Fig. 4C, D). Increased phosphorylation of the RPS6 indicates hyperactivation of mTORC1 in both pathologies (Fig. 5).

## Discussion

Molecular alterations, including different proteins and signaling pathways, associated with aberrant S-nitrosylation were found in both *Shank3* (ASD model) and P301S (AD model) mutant mice in this study. The SNO-related enrichment of synaptic-associated processes, serotonin 2A receptor (HTR2A) signaling, regulation of the cytoskeleton-related processes, and mTOR signaling were found to be shared between the two mouse models. These findings may provide mechanistic insight into the changes occurring to the S-nitroso-proteome that potentially can lead to the neuropathology.

NO is a multifunctional signaling molecule, that takes part in the regulation of neuronal and synaptic functions [29, 33, 37]. NO affects cellular signaling through cyclic GMP formation, proteins S-nitrosylation (SNO), tyrosine nitration, and *S*-nitrosoglutathione (GSNO) formation. SNO is a reversible NO-mediated posttranslational modification of cysteine thiols of proteins that modulate cell signaling pathways, neuronal functions, and synaptic plasticity [29, 30, 33, 37–41]. SNO occurs in different neuroanatomical regions, including the cortex, hippocampus, and striatum [42]. It contributes to multiple physiological and neuropathological processes. Thus, recently, we have shown a reprogramming of the S-nitroso-proteome during the aging process [37] and in response to arsenic exposure [43]. Further, we found significant sex differences in the NO and SNO-related biological functions in the cortex [33]. SNO of various proteins has been implicated in brain disorders, such as ASD [29, 41, 44], AD [30, 39, 41, 45, 46], Parkinson’s disease [41, 47, 48], Huntington’s disease [41, 45, 46, 48, 49], schizophrenia [41, 50], and other diseases. In the case of ASD, we suggest that aberrant SNO signaling of key proteins leads to synaptic deficits that converge into behavioral deficits in the *Shank3* mutant mice [29]. In the case of AD, it is still not clear how SNO affects the phenotype in the juvenile mice because at the age of 6–8 weeks there are no neuropathological features of AD. However, identification and characterization of the proteins that are S-nitrosylated in these mice might be an indicative for early diagnostic/pathological biomarkers of AD.

In our study, gene ontology (GO) analysis of the SNO proteins in ASD and AD mutant mice revealed enrichment of synaptic-associated processes. In particular, modulation of synaptic transmission, regulation of trans-synaptic signaling, synaptic vesicle fusion, and recycling appeared to be enriched in the mutant mice (Fig. 2C). Several studies have reported that precise control of synaptic functions and connectivity is crucial for maintaining normal brain functioning and the breakdown of these functions might be attributed to both ASD and AD pathologies [51–56]. Considering the involvement of protein SNO in the enrichment of synaptic processes in both mouse models in our experiments, it is logical to suggest that aberrant SNO signaling may lead to synaptic dysfunction that might contribute to the pathogenesis of ASD and AD. However, future validation studies should be conducted to confirm it.

Enrichment of “HTR2A signaling in the nervous system” (Fig. 2C) was also found in both models. HTR2A is highly abundant in the mammalian cortex, controlling multiple cognitive functions. The serotonergic system has been implicated in several neuropathological and neuropsychological conditions including mood disorders, schizophrenia, ASD, AD, and other brain pathologies [57–60]. We suggest that altered SNO signaling under neuropathological conditions might affect the neuromodulatory system, including the serotonin system, and subsequently facilitate the pathogenesis of ASD and AD.

It is worth mentioning that our analysis indicated enrichment of “regulation of cytoskeleton proteins” (Fig. 2C). The cytoskeleton is essential for proper neuronal functioning, axon guidance, and synapse formation [61]. Growing evidence suggests that abnormalities of cytoskeleton-associated proteins might result in detrimental effects contributing to neurodevelopmental disorders such as ASD, intellectual disabilities, and neurodegeneration [61–64]. It can be suggested that SNO-related abnormalities in the cytoskeletal proteins represent another pathogenic mechanism shared between ASD and AD.

Importantly, our systems biology analysis revealed the enrichment of the “mTORC1 upstream signaling pathway” by a subset of SNO proteins that were exclusively found in ASD and AD mouse models (Fig. 5). This indicates the possibility of altering the mTOR signaling as a result of aberrant SNO of specific proteins during neurodevelopment and neurodegeneration. mTOR is a highly conserved serine/threonine kinase, which plays an essential role in multiple physiological functions in the central nervous system, including neuronal differentiation, proliferation (neurogenesis), survival, dendritic arborization, synaptic formation, axonal regeneration, and dendritic spines growth via the regulation of protein synthesis that occurs through the phosphorylation of at least two major downstream targets; the p70 ribosomal protein S6 kinase and eukaryotic translational initiation factor 4E-binding protein 1 (eI4E-BP1) [65–67]. Thus, given the importance of the mTOR, it is not surprising that pathological hyper- or hypo-activation of this signaling pathway is potentially associated with a spectrum of neuropathologies, such as abnormal neuronal development, intellectual and learning disabilities, seizures, mental retardation, and neuronal degeneration [67–70].

Our work showed that the tuberous sclerosis complex 2 (TSC2) was S-nitrosylated in the *Shank3* model of ASD but not in WT mice (Figs. 2C and 5). TSC2 is an upstream master negative regulator of the mTOR activity. It forms a heterodimeric complex with TSC1 that suppresses mTORC1 activity through inhibition of the small GTPase Rheb, an essential activator of mTORC1 [71, 72]. The inhibitory effect of TSC2 on mTOR signaling is known to play a critical role in axon guidance, myelination, synaptic plasticity, and other neuronal processes [71]. Meanwhile, it is accepted that overactivation of the mTOR signaling contributes to the pathology of ASD [66, 73, 74]. Following a previous study [75], we suggest that SNO of TSC2 would result its autoubiquitination and in impaired TSC2/TSC1 dimerization, leading to a constitutive overactivation of mTORC1 that would enhance the severity of ASD phenotypes. This hypothesis is consistent with the findings of Reith et al. who showed that loss of Tsc2 in Purkinje cells may result in autistic-like behavior in mice. To further confirm the impact of SNO on mTOR signaling in the ASD mouse models, we quantified the phosphorylation levels of a major substrate of mTORC1, RPS6, that is widely used as a marker for neuronal activity and a readout of the mTORC1 activity [76]. The analysis showed increased RPS6 phosphorylation in the *Shank3* mutant mice compared to the WT indicating increased activity of mTORC1, likely in response to the altered SNO signaling.

Our systems biology analysis revealed elevated SNO of WNT11 and Frizzled10 in the P301S mutant mice (Figs. 2C and 5). These data are consistent with our previous studies showing that disruption of the WNT signaling is implicated in AD pathogenesis [30, 77]. Interestingly, it has been found that the WNT signaling pathway can mediate mTORC1 activity through the inhibition of GSK3β, a crucial regulatory kinase known to suppress mTORC1 by phosphorylating and activating TSC2 [78–80]. Increasing evidence [81, 82] points to the pivotal role of mTOR in multiple processes linked to AD, such as synaptic plasticity, aging, autophagy, long-term memory formation, etc. [83–85]. Some reports have indicated that reduced or increased mTOR signaling is associated with neurodegeneration [81, 82, 86]. These data prompted us to suggest that S-nitrosylation of the components of the WNT signaling pathway, such as WNT11 and Frizzled10, mediates dysregulation of this pathway resulting in altered mTOR signaling, which in turn contributes to AD pathology. Similar to the *Shank3* mutant mice, WB analysis revealed elevated levels of RPS6 in the P301S mutant mice compared to their WT counterparts, suggesting hyperactivation of mTORC1 in the AD mouse model.

Taken together, our results indicate that SNO-mediated abnormal activation of the mTOR signaling is involved in the pathogenesis of both ASD and AD. However, further validation studies are needed to investigate the direct effect of SNO on this pathway in these pathologies.

A total of 51 proteins showed to be S-nitrosylated in both mouse models. These included proteins such as Gap43, Camd2, P2rx3, Itpr2, and Nyap1. Gap43 is a neuron-specific calmodulin-binding protein that is thought to play a key role in axonal growth, neurogenesis, neuroplasticity, and synaptic transmission [87–90]. Altered expression of Gap43 was observed in both ASD and AD [90–92]. Camd2 is a synaptic cell adhesion molecule engaged in synapse organization, formation, and neuronal development [93–96]. Abnormal expression of this protein has also been proposed to contribute to the pathology of ASD and AD [95, 97, 98]. P2rx3 is an ionotropic ATP receptor that is mainly expressed in sensory afferent neurons and can functionally affect sensory transduction [99]. The accumulating evidence suggests a correlation between the changes in P2rx3 expression and the development of ASD and AD [100, 101]. Itpr2 has a centralized role in the processes of pruning improper synapses necessary for maintaining intact brain functioning [102]. Previous data suggest that deficiency in the Intpr2 is associated with both ASD and AD [102–104]. Nyap1 belongs to a family of phosphoproteins termed neuronal tyrosine-phosphorylated adaptor for the PI3-kinase (NYAP). Nyap1 is mainly expressed in the developing neurons and showed to play a pivotal regulatory role in neuronal morphogenesis, brain size, and neurite outgrowth via simultaneous activation of PI3K and the recruitment of the downstream effector, WAVE complex, to the PI3K [105]. It has been documented that disruption in Nyap1 also contributes to the pathogenesis of ASD and AD [105–107]. In line with these data, we suggest that aberrant SNO signaling induces functional changes to these proteins, affecting different processes and pathways related to neurodevelopment and neurodegeneration.

Our study showed that the SNOTRAP-based MS approach combined with large-scale systems biology analysis facilitates the global profiling of the SNO-proteome in both pathologies. Future studies of the effects of S-nitrosylation on these proteins may help to unravel the neuropathological mechanisms of ASD and AD. Finally, it is important to highlight that there are no data to indicate that such SNO-related abnormality is for all ASD and AD cases. Therefore, we emphasize that the shared mechanisms are specific to the two mutations investigated in this study.

In conclusion, our findings showed that the SNO signaling is altered by both mutations in *Shank3* and *Mapt* genes. In both datasets, SNO targets a wide range of proteins implicated in the regulation of neurodevelopment and neurodegeneration. Remarkably, S-nitrosylation of many of these proteins could be involved in the pathogenesis of both ASD and AD. Several signaling pathways and biological processes affected by SNO were found to be common to both pathologies. These included synaptic-associated processes, HTR2A signaling, PKA and calcium-mediated signaling, regulation of the cytoskeleton-related processes, and mTOR signaling. These proteins and pathways might serve in the near future as drug targets for the treatment of ASD and AD.

## Supplementary information

Supplementary Information

Supp. Table 1

Supp. Table 2
