# Supplementary material for: Proteomics of autism and Alzheimer’s mouse models reveal common alterations in mTOR signaling pathway
Source: Transl Psychiatry. 2021 Sep 17;11:480. doi: 10.1038/s41398-021-01578-2 (PMC8448888; doi:10.1038/s41398-021-01578-2)
Supplement: Supplementary file 1 — Supplementary Information [file 41398_2021_1578_MOESM1_ESM.docx]

**Supplementary Tables:**

Table 1: IDs of SNO-proteins in the different groups.

Table 2: System biology analysis of the SNO proteins in ASD and AD
